# Dr. Charles E. Boult

**Published:** 1914-01

**Authors:** 


Dr. Charles E. Boult, Buffalo, 1892, died at his home in Honeoye Falls, suddenly, Nov. 7, aged 54. Death is ascribed to heart disease. He was president of the local Board of Education and a coroner's physician of Monroe County.

